# The Vertebrobasilar Trunk and Its Anatomical Variants: A Microsurgical Anatomical Study

**DOI:** 10.3390/diagnostics14050534

**Published:** 2024-03-02

**Authors:** Gervith Reyes-Soto, Julio C. Pérez-Cruz, Luis Delgado-Reyes, Carlos Castillo-Rangel, Bernardo Cacho Diaz, Gennady Chmutin, Renat Nurmukhametov, Galina Sufianova, Albert Sufianov, Vladimir Nikolenko, Rinat Sufianov, Evgeniy Goncharov, Nicola Montemurro, Manuel De Jesus Encarnacion Ramirez

**Affiliations:** 1 Unidad de Neurociencias, Department of Head and Neck, Instituto Nacional de Cancerología, Mexico City 04260, Mexico; 2 Laboratorio de Técnicas Anatómicas y Material Didactico, Escuela Superior de Medicina, Instituto Politécnico Nacional, Mexico City 01070, Mexico; 3 Departamento de Anatomía, Facultad de Medicina, Universidad Nacional Autónoma de México, Mexico City 04510, Mexico; 4 Department of Neurosurgery, Servicio of the 1ro de Octubre Hospital of the Instituto de Seguridad y Servicios Sociales de los Trabajadores del Estado, Mexico City 07760, Mexico; 5 Functional Neurosciences Unit, Mexico National Cancer Institute, Mexico City 07760, Mexico; 6 Department of Neurological Surgery, Peoples Friendship University of Russia, 103274 Moscow, Russia; 7 Department of Pharmacology, Tyumen State Medical University, 625000 Tyumen, Russia; 8 Department of Pediatric Neurosurgery of Federal Center of Neurosurgery, Federal Center of Neurosurgery of Ministry of Health of the Russian Federation, 625000 Tyumen, Russia; 9 Department of Neurosurgery, I.M. Sechenov First Moscow State Medical University (Sechenov University), 119991 Moscow, Russia; 10 Department of Human Anatomy, I.M. Sechenov First Moscow State Medical University (Sechenov University), 119991 Moscow, Russia; 11 Department of Petrovsky Russian Scientific Center of Surgery, 121359 Moscow, Russia; 12 Department of Neurosurgery, Azienda Ospedaliero Universitaria Pisana (AOUP), 56100 Pisa, Italy

**Keywords:** basilar artery, perforants, anatomy, vertebral artery, terminal branches, arterial trunk, laboratory, neurosurgery

## Abstract

Background: The trunk of the basilar artery has not been included in microanatomy studies. Anatomical variants of the perforant branches of the vertebrobasilar trunk and their relationship with neural structures are very important in surgical approaches. Surgical dissection for the treatment of vascular lesions requires a perfect knowledge of the microsurgical anatomy. Methods: We conducted a descriptive analysis of 50 brains, which were fixed with formalin at 10% for 2 weeks, and the arterial system was injected with colored latex. After microsurgical dissection, it was divided into three segments: the lower portion went from the anterior spinal artery to the anteroinferior cerebellar artery, the middle segment was raised from the upper limit of the lower portion to the origin of the superior cerebellar artery, and the upper segment ranged from the previous portion until the origin of the posterior cerebral artery. Results: The basilar artery had an average length of 30 mm. The average diameter at its junction with the vertebral arteries was 4.05 mm. The average middle segment was 3.4 mm in diameter and 15.2 mm in length. The diameter of the upper segment was 4.2 mm, and its average length was 3.6 mm. The average number of bulbar arteries was three, and their average diameter was 0. 66 mm. The number of caudal perforator arteries were five on average, with a diameter of 0.32 mm. We found three rare cases of anatomical variants in the vertebra–basilar junction. Conclusions: The basilar artery emits penetrating branches in its lower, middle, and upper portions. The origin of penetrating branches was single or divided after forming a trunk. However, we observed long branches from perforant arteries.

## 1. Introduction

Surgical procedures targeting the base of the skull, particularly in the posterior fossa, require a detailed knowledge of the spatial arrangement of the vertebrobasilar vascular trunk and its anatomical variations. Recent developments in minimally invasive microsurgery methods and endovascular treatments underscore the need for a deeper understanding of these anatomical relationships. This knowledge is essential not only for the effective execution of existing surgical techniques but also plays a crucial role in the development of new minimally invasive microsurgical approaches. This knowledge serves as a cornerstone in grasping the complex anatomical interplays involved in pathologies of the posterior fossa. With rare exceptions, the vascular supply to critical brain structures such as the bulb, pons, midbrain, and cerebellum is predominantly provided by the vertebrobasilar system, a fact that is well documented in the literature [1,2,3,4,5].

A comprehensive understanding of the pathological conditions affecting the posterior circulation is contingent upon familiarity with both the embryological development and the anatomical nuances of this vascular network [6,7,8]. Additionally, the therapeutic application of bypass surgery for vertebrobasilar insufficiency and the management of posterior fossa tumors in proximity to the basilar trunk have accentuated the necessity of recognizing anatomical variations within this region [9,10,11,12,13].

While the foundational work of authorities like Testut and Latarjet [14] and Williams et al. [15] has illuminated the basilar artery (BA)’s intimate association with the skull base as it ascends through the clivus, they have not delved into the morphometric variables that may be critical for surgical consideration [16,17]. Clinical accounts from Dandy [18] and Watt and McKillop [19] have further shed light on the surgical challenges and complications encountered in posterior fossa approaches, highlighting the potential for vascular injuries. Such complexities underscore the limitations of classical anatomical texts, which often omit the intricacies and variations that are paramount to surgical success.

The call for a more detailed and nuanced anatomical understanding is clear. Surgeons and clinicians require access to comprehensive morphometric data and a deeper appreciation of the variability in vascular anatomy to navigate the delicate procedures of the posterior fossa successfully, thereby minimizing surgical risk and improving patient outcomes [20]. However, numerous variations in the caliber and course of posterior fossa arteries have been noted and described [20,21], all of which have emphasized the precise neurovascular relationships and anatomical features of the arteries in this region [22].

The evolving landscape of neurosurgery necessitates a thorough understanding of the posterior fossa’s complex vascular structures. Surgical intervention in this area is not only technically challenging but also poses significant risks due to the dense concentration of critical blood vessels and neural structures [23]. The vertebrobasilar system, which supplies the majority of the posterior fossa, including the cerebellum, brainstem, and related structures, is of particular interest due to its intricate anatomy and the potential for life-threatening complications if damaged [24].

Recent advancements in surgical techniques, particularly in minimally invasive and endovascular methods, have underscored the need for a more nuanced understanding of these vascular structures. Such knowledge is not only crucial for safe navigation and manipulation during surgical procedures, but also plays a pivotal role in preoperative planning and postoperative management [23,25]. In the context of vertebrobasilar insufficiency and posterior fossa tumors, the significance of recognizing anatomical variants cannot be overstated [26]. Variations in the course and caliber of the vertebrobasilar arteries can profoundly impact surgical approaches and outcomes. This is further complicated by the presence of numerous anastomotic channels and collateral pathways, which may vary significantly from one individual to another [27].

Despite the wealth of knowledge contributed by earlier anatomists and clinicians, there remains a gap in our understanding of the morphometric and functional aspects of these vessels. Contemporary research methods, including advanced imaging techniques and computational modeling, offer new opportunities to explore these aspects in greater depth [28]. By integrating these modern tools with the rich historical knowledge base, we can gain a more holistic and practical understanding of the posterior fossa’s vascular anatomy. Furthermore, a detailed study of the embryological development of these structures will contribute to a more profound comprehension of their anatomical variations and potential implications in pathological conditions [29,30]. Such knowledge not only benefits surgeons but also enhances the precision of diagnostic imaging and the effectiveness of non-surgical interventions [31]. The vertebrobasilar vascular system’s role in posterior fossa pathology is both critical and complex [32]. As surgical techniques continue to evolve, the need for a comprehensive understanding of this system’s anatomy and variations becomes increasingly important.

### 1.1. Basilar Artery

**Figure 1:**
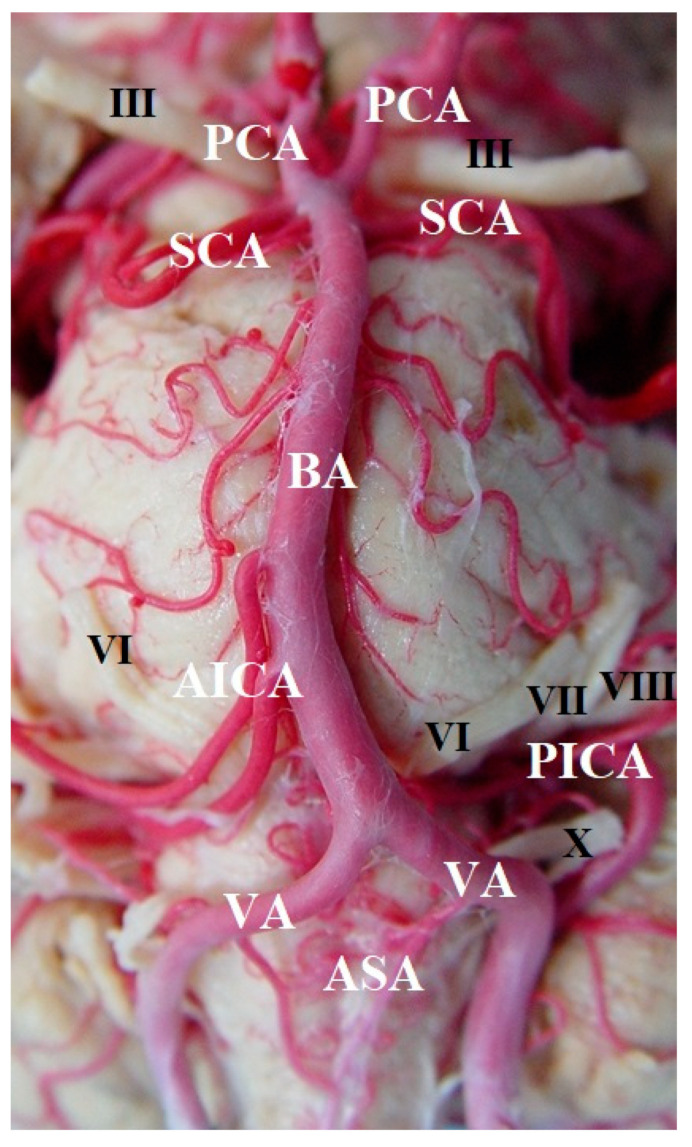
Vertebrobasilar arterial trunk: panoramic view. PCA, posterior cerebral artery; SCA, superior cerebellar artery; AICA, anterior inferior cerebellar artery; VA, vertebral artery; BA, basilar artery; ASA, anterior spinal artery; PICA, posterior inferior cerebellar artery; III, oculomotor nerve; VI, abducens nerve; VII, facial nerve; VIII, vestibulocochlearis nerve; X, vagus nerve.

The development of the BA arises from the fifth week of fetal development from the two longitudinal neural arteries, which are located on both sides of the developing rhombencephalon, slowly approaching each other until they fuse in the midline. The definitive BA is thus the result of the craniocaudal fusion of the two dorsal longitudinal neural arteries. This typically occurs between the height of the primitive trigeminal and hypoglossal arteries [33]. The caudal border of the definitive BA is plexiform for a considerable period of time, probably as a consequence of the presence of fenestrations at that location. By the sixth week of fetal development, the internal carotid artery provides blood flow for the anterior and posterior circulation [34]. The embryonic communications interconnecting these two circulations gradually return as the BA forms. They may persist as posterior communicating arteries. The developing vertebrobasilar system then assumes the dominant flow to the posterior fossa. The cerebellum develops relatively late in embryonic life compared to the cerebral hemispheres in the fourth and fifth months of fetal development. The superior cerebellar arteries will be the first to appear, followed by the anteroinferior cerebellar arteries. The posteroinferior cerebellar arteries are the last to develop. The great variability in these vessels is related to the relatively late persistence of the primordial vascular plexus in the posterior cerebral region (Figure 1).

The basilar extends superiorly from its origin next to the bulbopontine junction to its terminal bifurcation into the two posterior cerebral arteries. In 92% of cases, the terminal bifurcation of the BA is located in the interpeduncular cistern adjacent to the dorsum sellae or in the suprasellar cistern below the level of the floor of the third ventricle. The BA normally measures 32 mm in length and 3–4 mm in diameter [35].

The BA has labyrinthine, perforating (little studied), cerebellar, and hemispheric cerebral branches [36]. The BA may have some anatomical variants: The anterior inferior cerebellar artery (AICA) may share a common origin with the posterior inferior cerebellar artery (PICA) or originate from a single trunk, forming in this case the AICA-PICA trunk. The PICA, which usually originates from the vertebral artery, may vascularize the entire territory of the AICA, with the opposite also occurring on some occasions. An accessory AICA can supplement or replace part of the normal distribution of the AICA, a fact that is identified in 20% of anatomical dissections. Multiple superior cerebellar arteries (SCAs) are also frequent: a double SCA may be present in 8% or a triple SCA in 2% instead of a single dominant trunk [37]. Segmental hypoplasia of one or more of the vertebrobasilar components is not uncommon. If there is a fetal origin of the posterior cerebral artery (PCA) supplied from the anterior circulation, the BA appears to end up bifurcating into the SCAs. Duplication of the BA is associated with duplication of the pituitary gland in rare cases [2,38].

### 1.2. Vertebral Arteries

Embryological development: In the fourth week of fetal development at the 4 mm stage, the cranial and caudal divisions of the primitive internal carotid artery are already present. The two longitudinal neural arteries, two dorsally located parallel plexiform arterial arcades, appear around day 29 [30]. By the fifth week of fetal development, the vertebral arteries develop from plexiform anastomoses between the seven embryonic cervical intersegmental arteries. At this stage, the longitudinal neural arteries begin to join along the faces of the developing rhombencephalon. Transient anastomoses also develop between these vessels and the primitive carotid arteries. The most cephalic of these anastomoses, called carotid–basilar anastomoses, is the trigeminal artery. The longitudinal neural arteries initially receive blood supply from above by the trigeminal arteries, and from below by the cervical segmental arteries [31]. Each vertebral artery can be divided into four segments: (a) segment V1 (extraosseous), (b) segment V2 (foraminal), (c) segment V3 (extra-spinal), and (d) segment V4 (intradural). In addition, the branches of the vertebral artery have cervical, meningeal, and intracranial relationships.

There is significant variability in the relative size of both vertebral arteries. The left vertebral artery is dominant in most cases. In 25% of individuals, the right vertebral artery is larger than the left. The existence of a common trunk of the PICA and the AICA is a normal and frequent variant. Occasionally, a common trunk for posteroinferior cerebellar arteries may be seen. In 0.2% of cases, the vertebral artery terminates in the PICA; in this arrangement, the vertebral artery is small, and the contra-lateral provides most of the posterior fossa blood flow [34]. In 5–18% of cases, there is an extradural origin of the PICA; in this case, the PICA originates from the intracranial vertebral artery instead of from the extradural segment.

The PICA in this case curves upward through the foramen magnum to vascularize the amygdala and the inferior segments of the cerebellar hemispheres. In very rare cases, the PICA may originate as low as C1 and C2 [35]. In about one-third of cases, the PICA originates normally from the intradural vertebral artery, but its caudal loop extends a variable distance below the foramen magnum. A duplicated PICA is identified in 2% of anatomic dissections. In this case, the PICA arises as two or more vessels rather than as a single dominant trunk [36]. The vertebral artery has an anomalous origin in 5–6% of cases. The origin of the vertebral artery in the aortic arch is the most common anomaly, being present in approximately 5% of angiographies. An anomalous origin of the PICA is uncommon. The PICA may arise from the posterior meningeal artery or the internal carotid artery [23]. Origin of the posterior meningeal artery in the PICA has also been reported [36]. Bifid or duplicated origin and fenestration of the vertebral artery have also been published [30]. Duplication involves a vessel having two origins that follow a more-or-less parallel course for a variable distance. Fenestration occurs when a vessel has a normal origin and position but includes a double lumen in part of its course. Both duplicated and fenestrated vertebral arteries are rare anomalies, being found in less than 1% of anatomical dissections [36]. They probably represent a partial persistence of the embryonic plexiform ducts from which these vessels develop.

### 1.3. Carotid–Basilar Anastomoses

During the fetal development of the craniocerebral circulation, multiple vascular connections between the primitive carotid artery and vertebrobasilar circulation appear. With the exception of the extracranial proatlantal artery, these vessels are named according to the cranial nerve in relation. From top to bottom, they are described as follows: (a) persistent trigeminal artery, (b) otic artery, (c) hypoglossal artery, and (d) proatlantal artery.

Normally, these fetal anastomoses disappear as the posterior circulation develops over time. If a segmental anastomosis is not obliterated during development, persisting communication of the extracranial and intracranial circulation, it is called a basilar carotid anastomosis, according to the segment involved [36]. Two out of four embryonic vessels, the persistent hypoglossal artery and the proatlantal intersegmental artery, connect the cervical portion of the internal carotid to the basilar vertebral system [30].

The persistent trigeminal artery (PTA) creates a connection between the intracavernous segment of the internal carotid artery and the distal third of the BA. In some instances, the BA may be hypoplastic and not make contact with the trigeminal artery. The persistence of the trigeminal artery is observed in 1% of the general population, and in 30% it is associated with vascular malformations or cutaneous hemangiomas, with all types of congenital anomalies. The disturbance in embryonic vascular development must occur before 5 weeks, when the fetus is 14 mm long, and the trigeminal artery must have been isolated from the primitive carotid artery [30,34,36].

The persistent hypoglossal artery is the second-most frequent in carotid–basilar anastomoses, with a prevalence of 0.027 to 0.026% [30]. In this anomaly, the persistent embryonic vessel arises from the cervical portion of the internal carotid artery, usually between segments C1–C2, posteriorly with a posteromedial forward trajectory, reaching the anterior condyle foramen which enlarges it on its way to the intracranial circulation. The absence of posterior communicating vessels is associated with this variant.

The intersegmental proatlantal artery originates from the dorsal part of the internal carotid artery at its segments C2–C3, slightly below the origin of the persistent hypoglossal artery [34]. There are two types of intersegmental proatlantal arteries, in which type I is the most frequent and joins the vertebral artery after entering the foramen magnum, and type II joins the vertebral artery before entering the foramen magnum, with the latter frequently originating from the external carotid artery [39,40,41].

The primitive persistent otic artery is the rarest of the carotid–vertebral anastomoses; its existence has been questioned. It has been described as a branch from the petrosal segment of the internal carotid, directly medial and gaining access to the intracranial cavity through the internal auditory canal [39].

### 1.4. Objective

We performed a study by means of red latex injection in encephalons and later on, with the help of a surgical microscope, we describe and look for the anatomical variants with their morphometric description (length and diameter) in each of the vascular branches of the vertebrobasilar trunk. The anatomical division of the vertebrobasilar trunk is categorized into three distinct segments. The development of new minimally invasive neurosurgical techniques and posterior fossa cerebrovascular surgery require a thorough knowledge of the topographic relationship of the vertebrobasilar vascular trunk.

## 2. Materials and Methods

Our investigation is classified as a descriptive, cross-sectional, non-experimental study. We meticulously examined 50 human brains, focusing on their microsurgical anatomy. The primary aim was to elucidate the complex anatomical structures and variations within the vertebrobasilar trunk, offering valuable insights that are particularly relevant in the context of neurosurgical procedures.

### 2.1. Inclusion and Exclusion Criteria

Only brains aged from 18 to 80, of both genders, and from the Mexican population, which had been properly preserved, specifically fixed in formalin, and injected with colored latex for vascular highlighting were included in this study. Exclusion criteria were brains showing signs of damage, decomposition, or significant degradation affecting the accuracy of microsurgical analysis, brains that had undergone surgery or previous dissections, specimens that were not preserved using the formalin fixation method, specimens that did not undergo colored latex injection, and specimens not originating from the Mexican population to maintain the study’s demographic specificity and relevance.

### 2.2. Preparation and Fixation of Specimens

**Figure 2:**
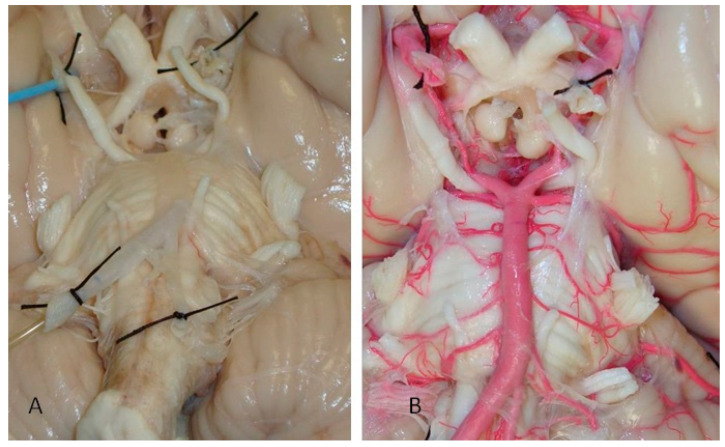
Injection and washing technique. (**A**) Arterial system during its irrigation with physiological solution. (**B**) Perfused arterial system with colored latex.

The brains were initially subjected to a thorough cleansing process, involving continuous irrigation with a 0.9% physiological solution. This step ensured the removal of any debris or clots that might interfere with the subsequent processes. Post-irrigation, the vertebral arteries of each brain were carefully perfused with a brightly colored red liquid latex. This latex perfusion was critical for delineating the intricate vascular structures during dissection. The perfusion process involved the precise placement of vascular clips at the posterior communicating segment and the PCA at the P2 level, ensuring targeted and efficient latex distribution. Following perfusion, the specimens were then submerged and fixed in a 5% formalin solution for a duration of 15 days. This fixation period was crucial for preserving the anatomical integrity of the brains for detailed microsurgical dissection (Figure 2).

Utilizing a high-precision Carl Zeiss OPMI™ (Oberkochen, Germany) surgical microscope, with magnification capabilities ranging from 6× to 40×, we performed intricate microsurgical dissections on each brain specimen. The dissections were meticulously documented using a high-resolution camera (with capabilities exceeding 8 megapixels), ensuring accurate visual records of the vascular structures and any observed variants. Measurements of the arterial diameters and lengths were precisely taken using a Mitutoyo Model CD-8″ CX digital Vernier (Kawasaki, Japan), calibrated to a resolution of 0.0005″/0.01 mm and utilizing millimeters as the unit of measurement.

### 2.3. Segmental Division and Categorization

For a comprehensive analysis, we divided the vertebrobasilar arterial system into three distinct segments:

- Inferior or caudal segment (CS): extending from the lower boundary of the anterior spinal artery (ASA) at its origin to the lower boundary of the AICA.
- Middle Segment (MS): spanning from the lower boundary of the AICA to the lower boundary of the SCA.
- Superior or rostral segment (RS): starting from the lower boundary of the SCA to the onset of the bifurcation of the BA.

In each segment, we meticulously analyzed the cerebellar arteries, the long pontine vessels, and the perforating vessels. This segmental approach allowed for a systematic and organized examination of the arterial structures, facilitating a clearer understanding of their distribution and relationships.

### 2.4. Variant Identification, Documentation, and Comparative Analysis

Variants identified during the dissection were documented separately. The focus was on accurately capturing the unique features and deviations from the typical anatomical presentations. These variants were not included in the comparative analysis with the non-variant brains, ensuring a clear differentiation and understanding of standard and atypical vascular structures. The collected data were analyzed descriptively using a combination of graphs and distribution tables. This approach provided a clear visual representation of the findings, aiding in the interpretation and comparison of the results. Our study’s outcomes were then compared with international studies that focused on individual vertebrobasilar segments. This comparative analysis aimed to contextualize our findings within the broader scope of existing global research, highlighting similarities, differences, and novel insights for surgical treatment.

This study was conducted in accordance with the Declaration of Helsinki and approved by the Ethics Committee of the Department of Head and Neck, Unidad de Neurociencias, Instituto Nacional de Cancerología, Mexico City, Mexico.

## 3. Results

The BA collateral branches originated in several directions during its course, which could be divided into three groups: the cerebellar arteries, the long pontine vessels, and the perforating arteries. The cerebellar arteries comprised the PICA, AICA, and SCA. The PICA originated in 10% of cases from the basilar trunk, and the AICA originated from the proximal half of the BA in 90% of cases; however, we observed that in all cases, the SCA originated from the distal half of the basilar trunk. The long pontine arteries comprised (a) the pontomedullary artery, (b) the posterolateral artery, and (c) the long pontine vessels, which are rostral or superolateral and caudal or inferolateral. The pontomedullary artery originates from the proximal half of the BA, with its direction towards the retro olivary fossa of the bulb. The posterolateral arteries arise from the distal half of the BA, proximal to the SCA. Finally, the long pontine vessels arise from the distal half of the BA and terminate in the vicinity of the trigeminal nerve.

The BA, from its origin near the pontobulbar junction to its termination near the pontomesencephalic junction, had an average length of 30 mm. The average diameter at the vertebral junction was 4.05 mm, and the average diameter at birth of the AICA was 3.5 mm, with average segment length of 17.1 mm. The MS had an average diameter of 3.4 mm and segment length of 15.2 mm. The RS had an average diameter of 4.2 mm and average length of 3.6 mm. The number of pontobulbar arteries was on average 3, with an average diameter of 0.66 mm.

AICA with inferolateral disposition to the VII and VIII pair, also found in 90% of the patients, had an average right diameter of 0.74 mm and an average left diameter of 1.02 mm. SCA had an average diameter of 4.2 mm, with the average bifurcation at 19 mm from its origin in ventral and dorsal. The number of caudal perforating arteries on average was 5, with an average diameter of 0.32 mm. The P1 segment averaged 7.6 mm in length, with a diameter of 2.3 mm, emitted approximately six branches, and included 95% of the thalamoperforating arteries.

The perforating branches originated directly from the BA, but also occasionally originated from its collateral branches. The perforating branches ranged in number from 8 to 19, and in diameter from 170μm to 840 μm. According to their position, origin, course, branching, relationship, and site of penetration, they were divided into three segments as discussed above (Table 1).

### 3.1. Inferior or Caudal Segment

**Figure 3:**
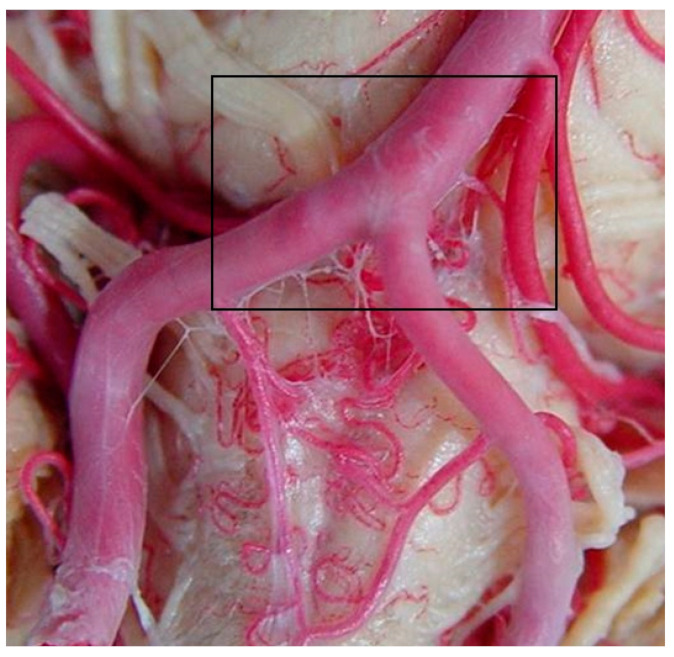
Caudal segment of the BA.

The inferior or CS includes the range from the inferior border of the anterior spinal artery to the inferior border of the AICA (Figure 3). The perforating arteries arise from the posterior surface of the BA, descend through the basilar sulcus, and enter the foramen cecum, where the junction of the pontomedullary sulcus and anterior median sulcus is located. The caudal perforators vary in number from one to seven, and in diameter from 170 to 630 μm; the average number was four, and the absence of perforators on the right side was observed in 15 (30%) brains and on the left side in 22 (44%). In this case, it was also observed that there was a contralateral common trunk that provided irrigation to this area.

In the caudal perforating arteries, we observed that they originated individually or as common trunks, where they subsequently emitted terminal branches. The average number of individual caudal perforators was zero to three for each BA, and their diameter was 100–390 μm; we observed an absence of individual perforators in 15 (30%) on the left side, and on the right side in 20 (40%). The existence of individual trunks was present in 31 (62%) brains on the right side and 34 (68%) on the opposite side. The diameter of the common trunks ranged from 530 to 550 μm. Common trunks originating from the lower segment branched individually, usually after entering the foramen cecum (FC).

The caudal perforating arteries almost always originate from the BA; however, we observed that in four (8%) brains their origin was from the left AICA, and in nine (18%) of them it was contralateral. Another origin observed was from the pontobulbar artery as collateral branches or as terminal branches of the same; specifically, from the right side in three (6%) cases and from the left side in eight (16%).

**Figure 4:**
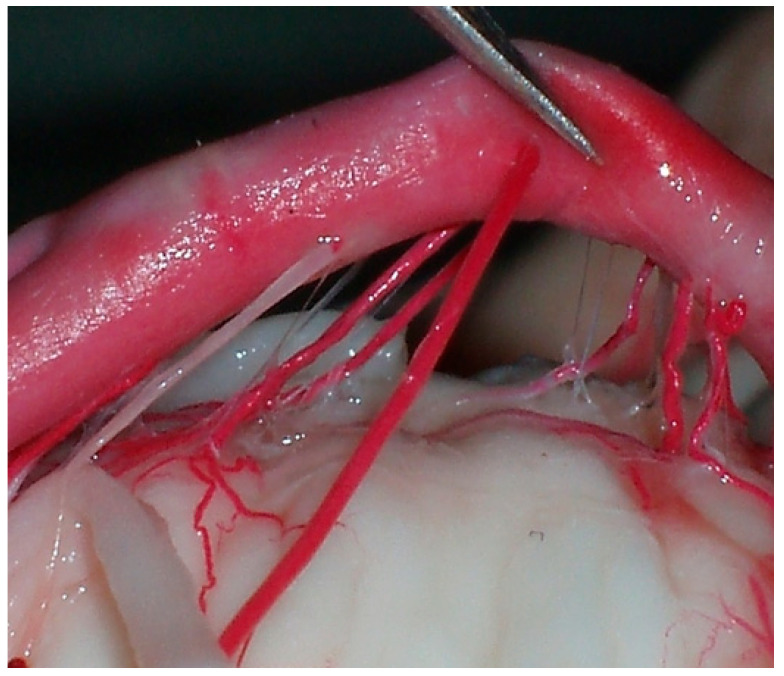
Caudal segment of the BA and related perforating arteries. Anastomoses were observed in 38 (76%) of all the brains, which ranged from three to nine in number per brain, and with diameters ranging from 135 to 190 μm. Anastomotic bridges were observed in 8 (16%) of encephalons between the middle and CSs of the BA, and also between the caudal perforators and the perforators of the vertebral artery in 13 (26%) encephalons; however, anastomoses between the caudal perforators were observed in only 2 (4%) encephalons.

Of the terminal branches that reached the FC in all cases, however, certain branches emitted three different kinds of branches towards the ventral surface of the medulla after emitting their branches towards the FC: (a) pyramidal branches that originated from the perforators in 18 (36%) on the right side, and 9 (18%) on the left side; (b) twin branches towards the rostral portion of the anterior medial sulcus of the medulla, which we observed in 7 (14%) on the right side and 3 (6%) on the left side; (c) finally, branches to the hypoglossal nerve unilaterally in 8 (16%) and bilaterally in 27 (54%) (Figure 4).

### 3.2. Middle Segment

The perforating arteries originate from the middle segment (MS), from the superior border of the AICA to the inferior border of the SCA. These branches originate entirely from the right and left posterior half of the BA. The more caudal perforators of the medial portion descend along the basilar sulcus or its borders, and the more rostral group ascends from along the same sulcus. The other perforators in the inferior segment were tortuous and curved; however, a different pattern was observed in this segment because their origin was tortuous and they subsequently entered the edge of the basilar sulcus radially. The medial perforators were observed bilaterally in all encephalons, and these varied in number from 5 to 8 with diameters of 210–640 mm. We observed the arrangement of perforators in individual trunks or branches as in the inferior segment. Common trunks were always present, ranging from 2 to 5 common trunks with average diameter of 420–960 μm; however, individual perforants with diameters of 210–640 mm were present in 34 (68%) on the right side and 46 (78%) on the left side. Thirty percent of all perforators originated from the MS of the basilar. In the remaining cases, some of them originated in relation to other branches of the BA. So, these originated within 5 mm of their origin from the AICA in 16%.

**Figure 5:**
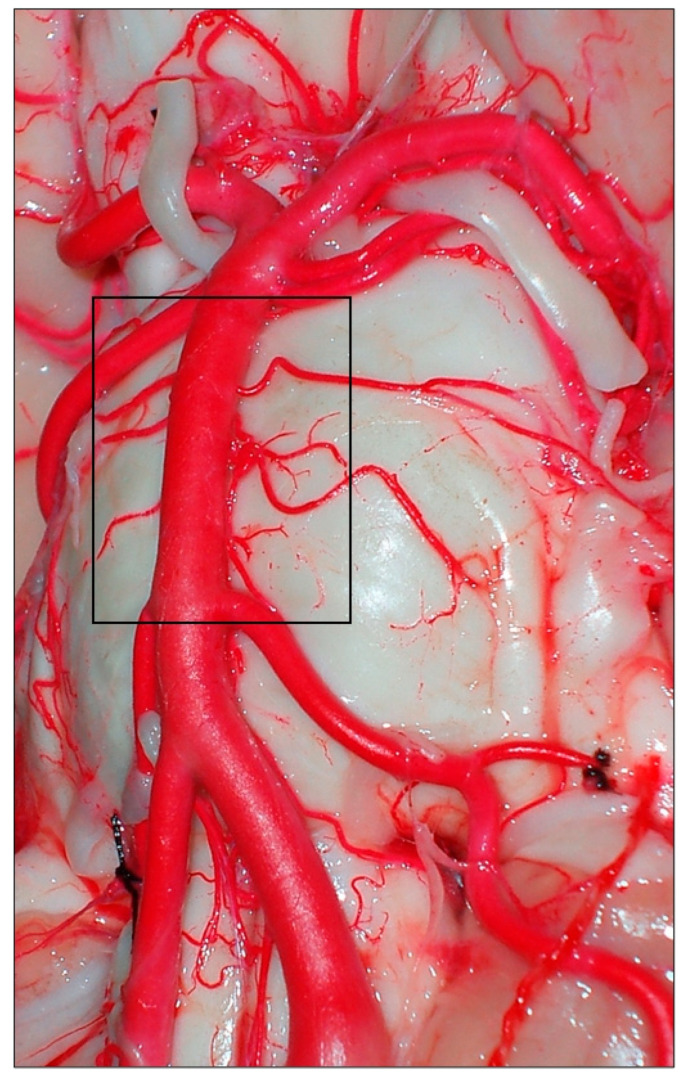
Brain stem, where the middle segment of the BA is observed.

They were observed to originate from the pontomedullary artery in 18%, and in 30% of cases they originated from the long pontine artery in its initial portion. One or more perforators originated as common trunks from the long pontine artery in 30% of the brains. However, common trunks originating with the antero-lateral arteries were less frequent, found in 15%. The presence of perforators with origin from the postero-lateral arteries was only observed in 10% (Figure 5).

Most of the mid-segment perforators originated between the origin of the AICA and the postero-lateral artery. In only 16.6% of the brains did they originate at the level of the AICA, and 25% originated below this level. The minimum distance between the origin of the AICA and the perforating branches was 1–6.8 mm (3.9 mm). Perforating branches were never observed to originate from the postero-lateral artery. The perforating arteries of the MS after their origin were directed rostrally and caudally, giving rise to their terminal and collateral branches. Several types of collateral branches can be identified; however, their frequency of origin from the perforating branches varies considerably. While the pontomedullary artery originates from the perforators in only 5 (10%), the long pontine arteries originate from the perforators in 15 (30%). However, one or more perforators gave rise to anterolateral branches in almost all the brains examined. These branches ranged from one to three per perforating artery, and their diameter from 230 to 620μm. The anterolateral caudal arteries were in close relationships with the abducens nerve, not only taking relation with it but also penetrating it in 10 (20%) of the brains.

Arterial complexes frequently exist within the MS of all perforators, which consist of a perforating branch, anterolateral arterial branch, and a branch of the sixth cranial nerve or a long pontine artery. These complexes were present in 25 (45%) right-sided hemispheres, and 32 (64%) left-sided hemispheres. Anastomoses were present in 37 (74%) hemispheres, but only one or two were observed in the same brain, between the middle and lower segment in 16 (32%). Anastomoses between adjacent an MS and its perforators were observed in 15 (30%) hemispheres. Right–left anastomoses as well as vascular connections between the rostral and middle portions were seen in only 7 (14%) (Table 2).

**Figure 6:**
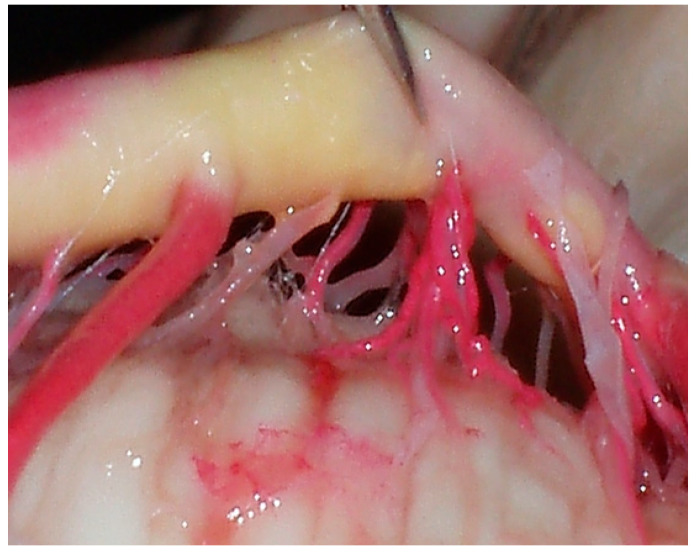
Middle segment of the inferior BA, perforating groups in a single arrangement or trunks.

The terminal branches of the MS penetrated the edges of the basilar sulcus. These were divided into long and short intrapontine branches, with a trajectory more or less close to the bridge raphe (Figure 6).

### 3.3. Superior or Rostral Segment

**Figure 7:**
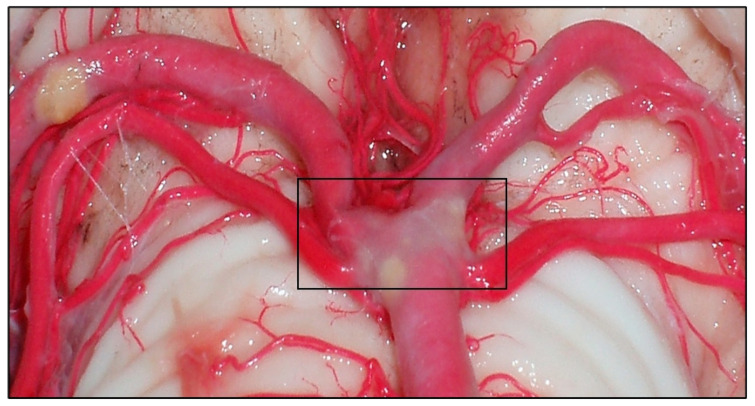
Rostral segment of the BA.

The perforating arteries of the RS range from the inferior border of the SCA artery to the beginning of the bifurcation of the BA (Figure 7). Their number ranges within 2–4, and their diameter within 220–530 μm. However, their absence was noted in 3 (6%) right-sided encephalons and 11 (22%) encephalons. In this segment, as in those previously mentioned, they originate as individual branches or as trunks. Individual perforators were present on the right side in 21 (42%) brains, and on the left side in 32 (64%) cases. The average diameter was 230μm. Common trunks were present in 30 (60%) on the right side and 25 (50%) on the left side. The diameter of the trunks ranged from 320 to 850 μm.

Only one to two perforating vessels originated from the BA in 40 (90%). However, they also originated with the same frequency from the SCA, and less frequently from the antero-lateral artery. Most of the arteries originated rostrally to the SCA artery; however, one of them originated at the level of the SCA origin in 35 (70%) encephalons and another one between the SCA and the posterolateral artery in 15 (30%) encephalons. Some others originated between the BA and PCA in 17 (34%). The distance between the sites of origin of the perforators and the SCA varied between 0.5 mm and 3 mm from their origin. The distance between the origin of the perforators and the bifurcation of the BA ranged from 0.8 mm to 4 mm. The RS perforators gave rise to anterolateral branches in 26 (55%) of the cases, with a trajectory near or along the pontomesencephalic sulcus.

**Figure 8:**
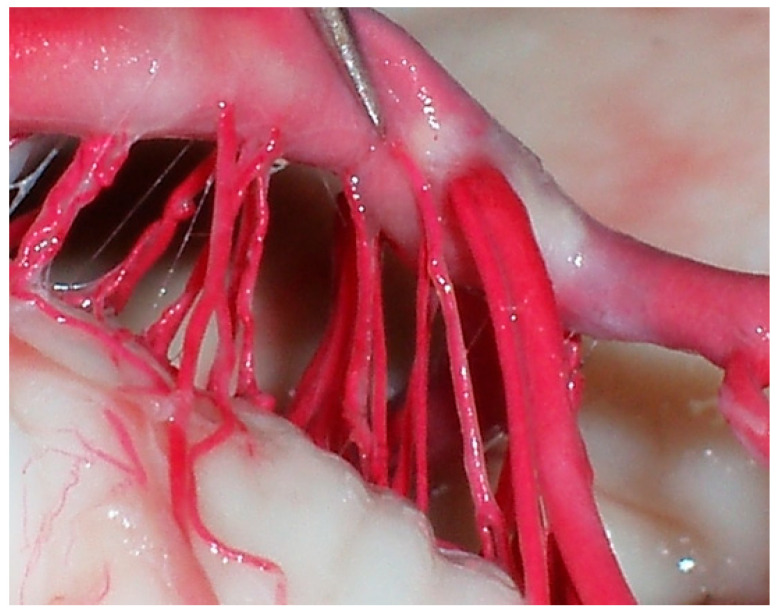
The upper segment of the BA and related perforating arteries.

We observed vascular anastomoses in 20 (40%) of all encephalons. Most of them were unilateral, but right–left anastomoses were present in only 2 (4%) of the hemispheres. The anastomoses were interconnected as follows: (1) between themselves, (2) with thalamoperforators, and (3) with SCAs. These anastomotic bridges ranged in diameter from 100 to 340 μm. The RS perforating arteries were located within the interpeduncular cistern, from where their collateral and terminal branches originated. The perforators entered from the most caudal part of the interpeduncular cistern in the most inferior part of the perforation site of the thalamoperforating arteries of the PCA (Figure 8).

### 3.4. Anatomical Variants

The anatomical variants found in our study comprise three: (a) persistent trigeminal artery, (b) fenestrated BA, (c) vertebral communicating artery; however, we found concomitant variants in the SCA, such as duplication in 15 (30%) brain; absence in 5 (10%) of the brains. Regarding the AICA, we found duplication in 13 (26%), predominantly on the left side in 3 (60%) of the blocks, and absence in only 1 (2%) of the blocks. The variants of the basilar vertebral system are described below.

#### 3.4.1. Right Persistent Trigeminal Artery

Its length was 48 mm with tortuous aspect, and this artery was divided in two segments: the inferior segment to the trigeminal artery and the distal segment to it, finding a distance from the vertebral junction to the inferior border of the trigeminal artery of 38 mm; and from the previous reference to the bifurcation of the basilar of 10 mm in the inferior segment we found a diameter of 4.1 mm; the segment proximal to the trigeminal artery was 3.5 mm, and its distal segment at the level of the basilar bifurcation was 4.3 mm. We also found hypoplasia of the AICA bilaterally with a diameter of 1.8 on the right side and 2.1 on the left side; in turn, we found that the superior cerebellar arteries had a greater diameter on the left side in relation to their counterpart: SCA diameter was 3.4 mm on the left and 2.4 mm on the right side.

**Figure 9:**
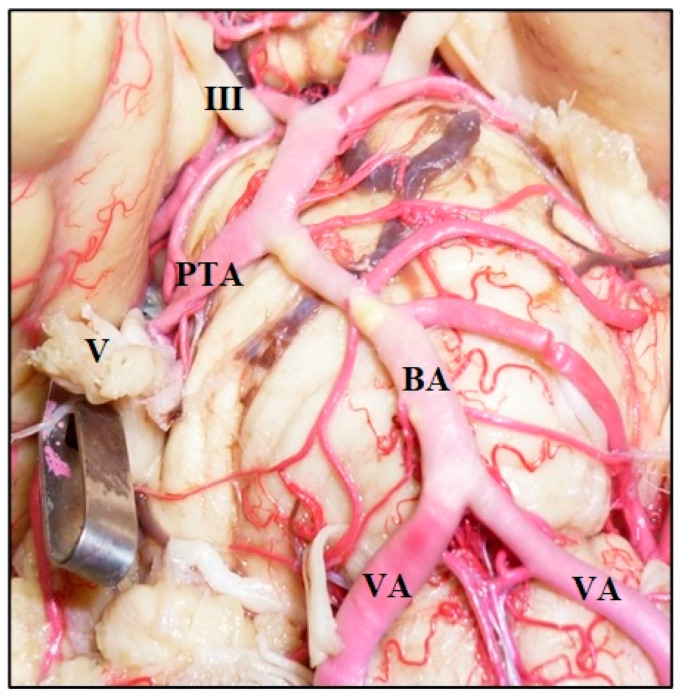
A right persistent trigeminal artery (PTA) and its relationship with the right trigeminal nerve (V). BA, basilar artery; III, oculomotor nerve; VA, vertebral artery.

The origin of the trigeminal artery was right-sided and 38 mm from the junction of the vertebral arteries, and its diameter was 4.5 mm. The system of perforating arteries of this variant is very complex because it presents in the lower segment a greater number of perforating trunks (four) and isolated perforators (five); the diameters of the trunks were in the range of 350–600 μm, and the diameters of the single perforating arteries were 200–340 μm. In the MS, we found fewer single trunks (two), and only three single perforating branches. The diameters of the trunks were larger with ranges of 400–850 μm, and the single perforators had ranges of 350–700 μm; we also found that perforators originated from the trigeminal artery in greater proportion as single perforators. In the rostral segment, we found five single trunks, there were three isolated perforators, trunk diameters were 350–500 μm, and isolated perforators were 140–230 μm in diameter. Posterior communicating arteries were present bilaterally. We did not find any structural alteration in the brain studied (Figure 9).

#### 3.4.2. Fenestrated BA

**Figure 10:**
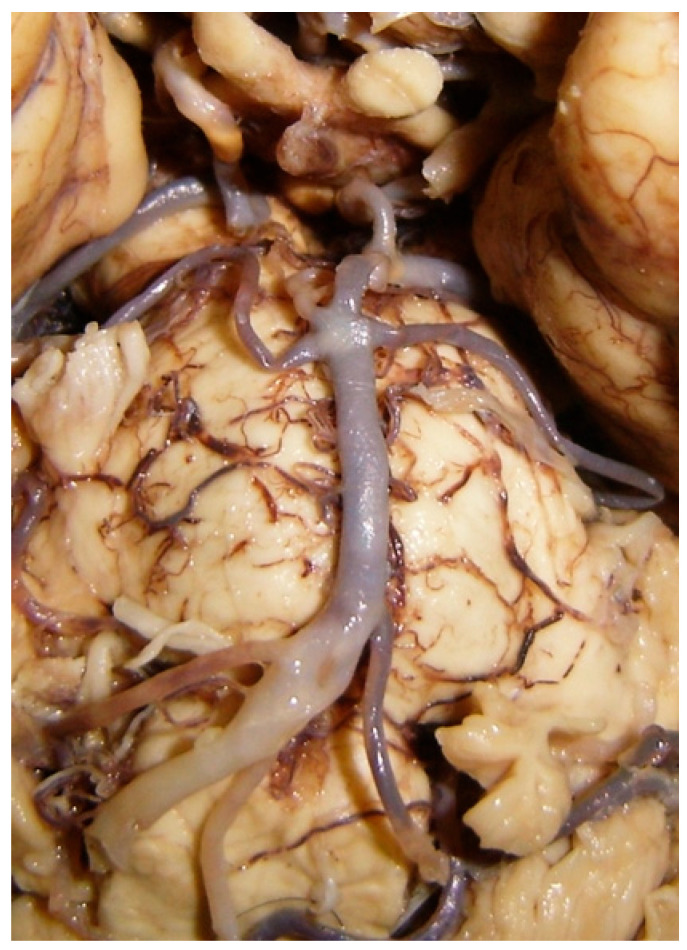
Fenestrated BA. It shows a widening and cleft of the right vertebrobasilar artery junction.

Its length was 39 mm from its origin to its bifurcation, finding a fenestration in the inferior segment, which widened the diameter of the inferior segment in relation to a fusiform aneurysm of the right-sided vertebral. Its inferior diameter was 4.8 mm, the MS was 3.5 mm in diameter, and the RS was 3.4 mm in diameter. We found that the anteroinferior cerebellar arteries were present bilaterally, with a diameter of 2.3 mm on the right side and 2.4 mm on the left side; in turn, we found that the left SCA was 3.4 mm in diameter and 3.8 mm on the right side. The system of perforating arteries in this brain presented three perforating trunks and four isolated perforators in the lower segment; the diameters of the trunks ranged within 240–500 μm, and the diameters of the single perforating arteries were 100–240 μm. In the MS we found 2 single trunks, and only 4 single perforating branches. The diameters of the trunks were larger, with ranges of 300–550 μm, and the single perforators were 150–400 μm in diameter. In the RSs, we found two single trunks, and two isolated perforants; furthermore, the trunk diameters were 180–450 μm, and isolated perforants were 170–280 μm in diameter. We did not find any structural alteration in the studied encephalon (Figure 10).

#### 3.4.3. Vertebral Communicating

**Figure 11:**
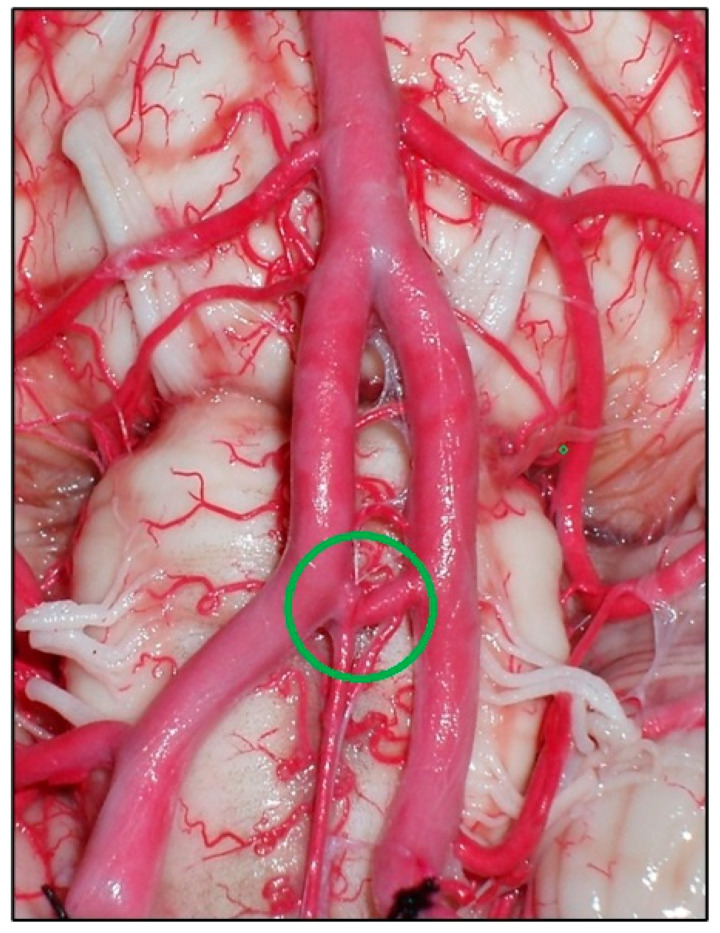
Vertebral communicating artery. The vascular bridge from which the spinal artery emerges is observed.

In this variant, we found that there was a real communication between the two vertebral arteries. Before forming the BA, this communicating artery emitted the anterior spinal artery. This vertebral bridge or vertebral communicant originated 18 mm from the vertebral junction on the right side and 16 mm on the left side, its diameter was 1.8 mm in its MS, and it had a length of 2.8 mm. The BA presented a length of 42 mm from its origin to its bifurcation. Its inferior diameter was 3.8 mm, the MS was 3.9 mm, and the RS was 3.7 mm. We found that the anteroinferior cerebellar arteries were present bilaterally, with a diameter of 1.9 mm on the right side and 2.2 mm on the left side, the superior cerebellar arteries on the left side 3.1 mm and 3.8 mm on the right side. The system of perforating arteries in this brain presented two perforating trunks and four isolated perforators in the lower segment, the diameters of the trunks were in ranges of 340–400 μm, and the diameters of the single perforating arteries were 140–180 μm. In the middle segment, we found four single trunks and only five single perforating branches. The diameters of the trunks were larger, with ranges of 350–480 μm, and the diameters of single perforators ranged within 250–370 μm. In the RSs, we found three single trunks, and five single perforants, the trunk diameters were 280–550 μm, and the single perforants’ diameters were 270–380 μm. We did not find any structural alteration in the studied encephalon (Figure 11).

## 4. Discussion

Posterior circulation develops in greater proportion during the first fourteen weeks of gestation. However, the vascular anatomy of the carotid circulation develops separately, showing anatomical variations in its final development. The spinal arteries and the vertebral artery are formed from the first six dorsal branches, given that the vertebral arteries establish communication with the anterior circulation by means of the BA, which arises from the consolidation of the longitudinal vascular channels. The adult BA is defined as the confluence of the vertebral arteries at the pontomedullary junction.

It is very common to find a dominant vertebral artery, usually right-sided, as was shown in this study finding this dominance. The anteroinferior cerebellar arteries are formed within the first centimeter of the birth of the basilar trunk, which frequently gives origin to the internal auditory and labyrinthine artery. Although the purpose of this study was not the vertebral arteries, a similar proportion was found according to the literature reviewed [39,41]. The paramedian, long circumferential, and perforating arteries provide irrigation to the pons and bulb. The SCA arises more distally, frequently in proximity to the bifurcation of the BA into posterior cerebral arteries.

This artery provides irrigation to the superior part of the pons, dorsal and lateral part of the midbrain, superior cerebellar peduncle, and superior or tentorial surface of the cerebellum [31]. The posterior cerebral arteries provide supply to the medial part of the temporal lobe and occipital lobe. The supply to the superior portion of the midbrain and thalamus comes from the perforating thalamic arteries, which arise directly from the distal part of the tip of the basilar at its P1 segment of the posterior cerebral. Eventually, the perforating thalamic arteries are odd and independently supply both thalami. When our results were compared with the findings of other authors [27,42], we found some differences to their perforators.

However, our description of the configuration, direction, and branching agrees with the reviewed authors. Concerning the total number of perforators, the discrepancies between our data and those reviewed were minimal, as in our study we found 8 to 19 perforators and in the reviewed studies these, on average, were 10 to 17. Some of the mentioned authors probably underestimated the number of perforators, mainly for the caudal and rostral groups, which are hidden between the BA and the basilar sulcus. Some other findings in this study have not been mentioned in the literature, such as the emergence of paramedian arteries from perforating trunks. This is of special relevance because of the relationship of the perforators with the hypoglossal nerve and external oculomotor nerve, as well as the site of the branches in the BA.

The perforating branches of the BA are usually divided into short and long intrapontine terminals [32]. The short vessels mainly irrigated the middle part of the pyramidal branches. The long branches supply the structures of the pontine tegmentum very close to the raphe nuclei and fourth ventricle, particularly the raphe nucleus, paramedian reticular formation, medial lemniscus, and external oculomotor nucleus. Marinkovic has attributed specific measurements of the number and diameter of perforators in different arterial segments. The caudal, middle, and rostral segments’ measurements resulted in 3.78 (3–6), 5.75 (5–8) and 1.5 (1–3) in number and 340 (170–430), 520 (210–740), and 300 (200–500) in diameter, respectively.

Vertebrobasilar disease accounts for 20% of all ischemic events. In general, it has a better prognosis than carotid artery ischemia, with an acute mortality of 3% [43]. However, approximately 14% of all patients with posterior circulation infarction have clear evidence of BA occlusion, and the prognosis is even more severe, with a mortality of 90% [44,45]. The first clinical study on vertebral occlusive disease was performed by Leyden in 1882, and his description was based on an analysis of cadaveric cerebral vasculature [46]. Currently, the use of magnetic resonance imaging (MRI) has been of great help in determining the vascular territories in patients with cerebral vascular events; however, surgical treatment is an important pillar in patients with vascular lesions in this region [47,48,49,50,51]. Therefore, a detailed knowledge of the anatomy of the region is required, with efforts to extract as many anatomical data as possible from the studies to determine the appropriate approach. Using the conventional techniques of MRI and angiography, it is difficult to appreciate the relationship between vascular and bony structures [45,46]. Three-dimensional tomography has allowed for the reconstruction of skull base structures with vascular, nervous, and bone relationships that have been of great help in surgery. However, precise knowledge of the anatomical variants and even the vascular territories involved are of great help to vascular neurosurgeons. Technological development has allowed for the use minimally invasive procedures for cerebral revascularization, the use of excimer lasers as support in the development of cerebral bypass with greater chance of success, and in giant aneurysms a combined management with coils or posterior clipping that normally would only be viable with flow diversion due to complexity or size [52,53,54].

Infratentorial aneurysms comprise between 2.5 and 15% of all saccular aneurysms. Of these, BA aneurysms account for 59% to 77%. BA aneurysms are frequently associated with fenestration of the BA [50]. Fusiform aneurysms may involve inferior or total segments of the BA. Saccular aneurysms can be located at the bifurcation of the BA, at the origin of the SCA, between the SCA and the P1 segment of the PCA; at the origin of the emergence of the lateral pontine artery; and in the middle portion of the basilar trunk, between the two long pontine arteries, at the site of origin of the anteroinferior cerebellar arteries [9,10,11,12]. Skull base approaches, including the temporopolar approach, anterior petrosectomy with Kawase technique, and the combined transpetrosal approach, are currently the standard surgical approaches for the treatment of BA aneurysm, replacing conventional approaches such as the pterional and subtemporal approaches [55]. These approaches allow for the conversion of a narrow and deep corridor of the skull base into a wide surgical passageway [56].

## 5. Conclusions

Detailed knowledge of the anatomy of the vertebrobasilar artery and its variants will allow for a better understanding that can be used in the treatment of vascular diseases or diseases involving its anatomy, such as skull base tumors. In our study, we describe that each of the perforators has a greater diameter and a greater number of trunks in relation to the branches individually. The development of new minimally invasive techniques in vascular and skull base surgery will, in the future, allow us to improve prognoses in patients with vascular insufficiency by bypass or complex aneurysms. This is why our study will certainly help in the development of these techniques.

## Figures and Tables

**Table 1:** Diameter of the arteries of vertebrobasilar trunk.

Artery	Average Diameter Left (mm)	Average Diameter Right (mm)
Vertebral	2.72 (1.5–4.5)	2.45 (1–4.0)
PICA	1.23 (0.8–2.5)	1.36 (0.7–3.0)
AICA	0.89 (0.2–1.5)	0.87 (0.2–1.5)
SCA	1.6 (1.3–1.9)	1.81 (1.4–2.2)
PCA	2.3 (1.6–3)	2.4 (2.2–2.6)

PICA, posterior inferior cerebellar artery; AICA, anterior inferior cerebellar artery; SCA, superior cerebellar arteries; PCA, posterior cerebral artery.

**Table 2:** Diameter and number of trunks with individual perforators’ details, and various measurements related to anatomical structures in different segments.

Segment	Number of Perforators	Diameter of Perforators (µm)	Number of Trunks with Perforators	Diameter of Arterial Trunks (µm)	Individual Perforators	Diameter of Individual Perforators (µm)
Upper Segment	2–4	220–530	1–2	320–850	1–3	75–490
Middle Segment	5–8	210–640	3–6	420–960	2–5	210–640
Lower Segment	1–7	170–430	0–5	530–550	0–3	100–390
Total	8–19	170–640	4–13	320–960	3	75–510

## Data Availability

The data presented in this study are available on request from the corresponding author.
